# ASPP2 Is a Novel Pan-Ras Nanocluster Scaffold

**DOI:** 10.1371/journal.pone.0159677

**Published:** 2016-07-20

**Authors:** Itziar M. D. Posada, Marc Serulla, Yong Zhou, Christina Oetken-Lindholm, Daniel Abankwa, Benoît Lectez

**Affiliations:** 1 Turku Centre for Biotechnology, Åbo Akademi University, Tykistökatu 6B, 20520, Turku, Finland; 2 University of Texas Health Science Center at Houston, Medical School, Houston, Texas, United States of America; Medical College of Wisconsin, UNITED STATES

## Abstract

Ras-induced senescence mediated through ASPP2 represents a barrier to tumour formation. It is initiated by ASPP2’s interaction with Ras at the plasma membrane, which stimulates the Raf/MEK/ERK signaling cascade. Ras to Raf signalling requires Ras to be organized in nanoscale signalling complexes, called nanocluster. We therefore wanted to investigate whether ASPP2 affects Ras nanoclustering. Here we show that ASPP2 increases the nanoscale clustering of all oncogenic Ras isoforms, H-ras, K-ras and N-ras. Structure-function analysis with ASPP2 truncation mutants suggests that the nanocluster scaffolding activity of ASPP2 converges on its α-helical domain. While ASPP2 increased effector recruitment and stimulated ERK and AKT phosphorylation, it did not increase colony formation of RasG12V transformed NIH/3T3 cells. By contrast, ASPP2 was able to suppress the transformation enhancing ability of the nanocluster scaffold Gal-1, by competing with the specific effect of Gal-1 on H-rasG12V- and K-rasG12V-nanoclustering, thus imposing ASPP2’s ERK and AKT signalling signature. Similarly, ASPP2 robustly induced senescence and strongly abrogated mammosphere formation irrespective of whether it was expressed alone or together with Gal-1, which by itself showed the opposite effect in Ras wt or H-ras mutant breast cancer cells. Our results suggest that Gal-1 and ASPP2 functionally compete in nanocluster for active Ras on the plasma membrane. ASPP2 dominates the biological outcome, thus switching from a Gal-1 supported growth-promoting setting to a senescence inducing and stemness suppressive program in cancer cells. Our results support Ras nanocluster as major integrators of tumour fate decision events.

## Introduction

Cancer associated Ras isoforms K-ras4A, K-ras4B, H-ras and N-ras are membrane anchored small GTPases that relay extracellular signals. Ras signalling can influence opposing cellular events, such as cell proliferation or differentiation, as compared to senescence or apoptosis [1–3]. The four Ras isoforms share a highly-conserved sequence with the exception of the C-terminal region called the hypervariable (HVR) region, which is isoform specific [4].

HVR-instructed specific activities of Ras proteins are supported by a number of observations. For example, different isoforms activate the effectors to different extents [5–7]. Moreover, recent data suggest that Ras isoforms specifically affect the fate of stem cells, with H-ras driving differentiation, N-ras being apparently neutral and K-ras4B (hereafter K-ras) stimulating self-renewal [8]. Related to this, K-ras instructs stemness in cancer cells, making it a more potent tumour initiator as compared to H-ras [9].

The HVR furthermore directs Ras isoforms to laterally segregate in the plasma membrane into distinct, non-overlapping nanoscale signalling hubs called nanoclusters [10–12]. The application of advanced quantitative imaging techniques, such as electron microscopy and fluorescence microscopic methods allows to investigate Ras nanoclustering in mammalian cells [12,13]. Nanoclusters are essential sites for effector recruitment and seem to integrate protein interactions that are required for Ras isoform specific signalling output [14,15]. Therefore, understanding the architecture of Ras nanocluster will help to resolve the apparent promiscuity of Ras proteins to interact with various effectors [16].

Nanocluster formation and signalling output can be regulated by nanocluster scaffolding proteins. Only a few Ras nanocluster scaffolds are known, including Galectin-3 and nucleophosmin specifically for K-ras [17,18], as well as Galectin-1 (Gal-1), which positively regulates GTP-H-ras, but negatively GTP-K-ras nanocluster [15,19,20]. Of note, Gal-1 was shown to divert Ras signalling to Raf at the expense of PI3K [21], illustrating that nanocluster scaffolds may play an important role in defining Ras isoform specific events. So far, no N-ras associated nanocluster scaffold is known.

Ras isoforms are mutated in the 30% of human cancers and are major drivers of tumourigenesis [22]. Cells have evolved safety mechanism to shut down overactive Ras. For instance, excessive MAPK signalling in fibroblasts initiates a permanent cell cycle arrest or even induction of apoptosis that depends on functional p53 [1]. In this context, the Apoptosis-Stimulating of p53 Protein (ASPP) family member ASPP2 is considered a key mediator in Ras oncogene induced senescence and apoptosis response [23]. In line with this, ASPP2 downregulation in cancer has been associated with increased tumourigenesis [24,25]. More recently, another member of the family, ASPP1, has been shown to coordinate with p53 in restricting the self-renewal capacity in hematopoietic stem cells [26].

Of note, the Ras/MAPK pathway engages ASPP2 on several levels. ASPP2 was reported to interact via its N-terminal ubiquitin-like (Ubl) domain with GTP-H-ras [27], and increase ERK phosphorylation. Phosphorylation of Ser826 in the proline-rich domain of ASPP2 by activated ERK induces ASPP2 translocation to the nucleus [3], where it interacts via its C-terminal domain with p53 to effect apoptosis [28–30]. It also mediates Ras-induced senescence by inhibiting autophagy in a p53-independent fashion [31]. ASPP2 functions might be modulated by its oligomeric state, as *in vitro* experiments indicated that ASPP2 can form homodimers [28].

Here, we show that ASPP2 promotes nanoclustering, effector recruitment and downstream signalling of constitutively active H-ras, K-ras and N-ras. This activity appears to reside in the α-helical domain of ASPP2. ASPP2 is therefore the first nanocluster scaffold of N-ras. Intriguingly, ASPP2 is able to antagonize the activity of Gal-1, which therefore abrogates Gal-1 promoted transformation of H-rasG12V-NIH/3T3 cells and mammosphere formation. These data suggest that ASPP2 competes on the nanoscale with Gal-1 to effectively switch the cell fate from proliferation to senescence.

## Materials and Methods

### Cell culture and DNA constructs

Human Embryonic Kidney 293-EBNA (HEK), BHK21, MCF-7, HS-578T and NIH/3T3 cells were cultured in Dulbecco´s modified Eagle´s medium (DMEM, Sigma-Aldrich, Helsinki, Finland). MDA-MB-231 cells were incubated in Roswell Park Memorial Instituted medium (RPMI, Sigma-Aldrich). Media were supplemented with 10% fetal bovine serum (not heat-inactivated in case of NIH/3T3 cells) and 1% L-glutamine. The cells were incubated at 37°C with 5% CO_2_. Transfections were performed with jetPRIME (Polyplus transfection, Illkrich-Graffen-staden, France), unless stated otherwise.

Plasmids pmGFP-H-rasG12V, pmGFP-K-rasG12V, pmGFP-N-rasG12V, pcDNA3-Gal-1, pcDNA3-Gal-1-C3S,L5Q,V6D,A7S (pcDNA-N-Gal-1), and mRFP-RBD with the Ras binding domain (RBD) of C-Raf were described before [11,15,19,32]. PmCherry-RasG12V constructs were generated by replacing mGFP from pmGFP-RasG12V with mCherry from pmCherry-C1 vector (Clontech Laboratories Inc., CA, USA) using NheI and BsrGI restriction sites. Plasmid pCMV-Sport6-ASPP2 was from Dharmacon (ref. MHS6278-202758068, Lafayette, CO, USA). Plasmids pcDNA3-ASPP2(1–360)-V5, pcDNA3-ASPP2(123–1128)-V5 and pcDNA3-ASPP2(1–1128)-V5 were a kind gift from Dr. Xin Lu [31].

### FRET-imaging using fluorescence lifetime microscopy (FLIM)

HEK cells were seeded on a 6-well plate with glass coverslips, and transfected with the donor alone (mGFP-tagged Ras constructs) in control samples, or together with the acceptor. Acceptors were mCherry-tagged Ras constructs in nanoclustering-FRET experiments (mGFP-: mCherry-plasmids at 1:3 ratio, 2 μg total plasmid), or mRFP-RBD in C-Raf-RBD-recruitment FRET experiments (mGFP-: mRFP-plasmids at 1:3 ratio, 2 μg total plasmid). The cells were further cotransfected with either 1.5 μg empty pcDNA (control) or pcDNA plasmids for expressing Gal-1, full length ASPP2, ASPP2(1–360) or ASPP2(123–1128) constructs, or the combination of Gal-1 or N-Gal-1 and either of the ASPP2 proteins (1:1 ratio, 3 μg total plasmid). After 48 h of transfection, coverslips were fixed with 4% PFA/PBS for 15 min and then washed with PBS, and coverslips were mounted with Mowiol 4–88 (Sigma Aldrich) on microscope slides.

The mGFP fluorescence lifetime was measured using a fluorescence lifetime imaging attachment (Lambert Instruments, Groningen, Netherlands) on an inverted microscope (Zeiss AXIO Ovserver.D1, Jena, Germany) as previously described [14]. Per condition, the fluorescence lifetime was measured typically for >40 cells from three biological repeats. The percentage of the apparent FRET efficiency (E_app_) was calculated using the measured lifetimes of each donor-acceptor pair (τ_DA_) and the average lifetime of the donor only (τ_D_) samples. The formula employed was Eapp = (1-τ_DA_/ τ_D_) x 100%.

### Immuno-electron microscopic analysis of Ras nanoclustering

Immunoelectron microscopy spatial mapping was performed as described previously [33,34]. Apical plasma membrane sheets were prepared from BHK cells transiently expressing mGFP-tagged oncogenic Ras with or without coexpressed ASPP2, fixed with 4% PFA/PBS and 0.1% glutaraldehyde, and labeled with 4.5 nm (diameter) gold nanoparticles coupled to anti-GFP antibody. Digital images of the immuno gold-labeled plasma membrane sheets were taken at 100,000× magnification in an electron microscope Jeol JEM-1400 (JEOL USA, Inc., Peabody, MA, USA). Intact 1 μm^2^ areas of the plasma membrane sheet were identified using ImageJ, and the (*x*, *y*) coordinates of the gold particles were determined as described. A minimum of 15 plasma membrane sheets were imaged and analysed for each condition. A bootstrap test constructed as previously described was then used to evaluate the statistical significance of differences between replicated point patterns [35].

### Western Blotting

For SDS-PAGE analysis, HEK cells were harvested in a buffer containing 50 mM dithiothreitol, 2% sodium dodecyl sulfate, 10% glycerol, 0.1% bromophenol blue and 10 mM Tris-HCl, pH 6.8. Proteins were first separated using SDS polyacrylamide gels (10%) and then electroblotted on nitrocellulose membrane (Perkin Elmer, Waltham, MA, USA). Membranes were immunolabelled using primary antibodies from the following sources: antibodies to phospho-T202/Y204 ERK (catalogue no. 9101), ERK 1/2 (no. 9102) and AKT (no. 9272) were from Cell Signaling Technologies (Danvers, MA, USA); phospho-T308 AKT (no. MAB7419) was from R&D Systems (Wiesbaden, Germany); Gal-1 (no. 500-P210) was from Peprotech (Hamburg, Germany); GFP (no. 3999–100) was from Bio-Vision (Milpitas, CA, USA) and β-actin (no. A1978) from Sigma Aldrich. ASPP2 (LX50.13) primary antibody was a kind gift from Dr. Xin Lu [36]. Horseradish peroxidase-conjugated chicken anti-mouse and goat anti-rabbit IgG secondary antibodies (cat. nos. sc-2954 and sc-2004) were from Santa Cruz Biotechnology (Paso Robles, CA, USA) Protein bands were detected with enhanced chemiluminiscence (Clarity Western ECL Substrate, Bio-Rad, Helsinki, Finland).

### Confocal Imaging

HEK cells were transfected with mGFP-RasG12V plasmid or/and the plasmids encoding ASPP2 proteins. After 48 hours, cells were fixed with 4% PFA/PBS for 15 min and immunostained, if required, using primary anti-ASPP2 antibody and a secondary Alexa-647 anti-mouse antibody. Cells were then mounted with Mowiol 4–88 on microscope slides. Cells were imaged using a Zeiss LSM 780 confocal microscope (Carl Zeiss AG, Jena, Germany) with a 63x NA 1.2 water immersion objective, using a 488 nm wavelength argon laser (2% of nominal power 35 mW) for mGFP (detection: 500–580 nm) and a 633 nm wavelength HeNe laser (detection: 638–758 nm). Bidirectional scanning and 8x line averaging was used on a 1024x1024 resolution with zoom adjusted according to the cell of interest. Images were analysed using ImageJ (National Institutes of Health, Bethesda, MD, USA).

### GFP Pull-down assay

To study the interaction between ASPP2 constructs and H-ras, HEK cells were cotransfected with plasmids encoding mGFP-H-rasG12V and full-length ASPP2, ASPP2(123–1128) or ASPP2(1–360) (H-ras:ASPP2 plasmids at 1:3 ratio, 2 μg total plasmid). After 48 h, cells were washed twice with PBS and incubated with 0.8 mM of the cross-linker dithiobis(succinimidyl-propionate) during 30 min. The reaction was stopped with incubation of 20 mM Tris in PBS pH 7.4 during 15 min. After two washes with PBS, cells were lysed in 200 μl of lysis buffer (25 mM Tris–HCl pH 7.5, 150 mM NaCl, 1 mM EDTA, 1% Triton, 1× Proteases Inhibitor cocktail) during 30 min and collected for centrifugation at 20,000× g for 10 min at 4°C. The supernatant was mixed with pre-washed GFP beads (Chromotek GmbH, Planegg, Germany) with dilution buffer (10 mM Tris–HCl pH 7.5, 150 mM NaCl, 0.5 mM EDTA, 1× Proteases Inhibitor cocktail). The mixture was incubated at 4°C for 2 h with gentle rolling and centrifuged at 2,500× g for 2 min. The supernatant was removed and the beads were washed twice with dilution buffer. The bound proteins were eluted with SDS-PAGE sample loading buffer (250 mM Tris–HCl pH 6.8, 20% glycerol, 4% SDS, 0.4% bromophenol blue, 10% β-mercaptoethanol) heated at 95°C for 10 min and loaded into 4–20% Tris–Glycine gels. After electroblotting, membranes were immunolabeled using primary-antibodies anti-ASPP2 and anti-GFP (3999–100, BioVision, Inc., Milpitas, CA, USA) and membranes developed as described under Western Blotting.

### Colony survival assay

NIH/3T3 cells stably expressing mGFP-H-rasG12V or mGFP-K-rasG12V were used [13]. 3,000 cells were seeded per well on a 6-well plate. When colonies started to be noticeable, cells were transfected with either empty pcDNA (control) or with 1.5 μg plasmid expressing Gal-1, ASPP2 or the combination of both (1:1 ratio, 3 μg total plasmid) using Lipofectamine 3000 (Thermo Fisher Scientific, Grand Island, NY, USA), or were incubated with 5 μM compactin. The colonies were allowed to grow for additional 3–4 days and then fixed with 4% PFA/PBS for 15 min, stained with 0.5% crystal violet in 10% ethanol for 15 min and washed with PBS to remove the excess of stain. The average colony area percentage was calculated using the “colony area” ImageJ plugin [37].

### Mammosphere formation assay

MCF-7, HS578T and MDA-MB-231 cells were first seeded in 6-well plates and transfected with either empty pcDNA (control) or with 1.5 μg plasmid expressing Gal-1, ASPP2 or the combination of both (1:1 ratio, 3 μg total plasmid) using JetPrime for MCF7 cells or Lipofectamine 3000 (Thermo Fisher Scientific) for HS578T and MDA-MB-231 cells [38]. 24 hours later cells were transferred to 48-well suspension culture plates (Cellstar, Grenier BioOne, Frickenhausen, Germany) at an initial density of 4,000 cells/well in serum-free media supplemented with 1x B27 (Gibco, Thermo Fisher Scientific), 25 ng/μl of Epidermal Growth Factor (Sigma) and 25 ng/μl of Fibroblast Growth Factor (Sigma). After 10 days of culture, mammospheres were analysed in an Evos FL microscope (Thermo Fisher Scientific). Spheres with a minimun size of 50 μm were counted.

### Senescence associated-β-galactosidase activity assay

Gal-1 and ASPP2 effect on MCF-7 cell senescence was studied by following senescence-associated (SA)-β-gal activity. Cells were transfected with plasmids encoding Gal-1, ASPP2 or the combination of both proteins. Cells were allowed to grow for 7 days, fixed and stained with the X-gal solution from the Senescence Cells Histochemical Staining Kit (Sigma-Aldrich, St Louis, CA, USA) according to the manufacturer’s protocol. Senescent cells were quantified under a light microscope Olympus BX60 (Olympus America Inc, San Jose, CA, USA) by counting SA-β-gal positive cells.

### Statistical analysis

Unless otherwise stated, statistical differences were determined using an analysis of variance (ANOVA) complemented by Tukey’s honest significance difference test (Tukey’s HSD) performed in GraphPad PRISM software. Statistical significance levels are annotated as ns, not significant; *, p < 0.05; **, p < 0.01; ***, p < 0.001; ****, p < 0.0001.

## Results

### ASPP2 increases oncogenic H-ras, K-ras and N-ras nanoclustering

ASPP2 was reported to bind to GTP-H-ras via its Ubl domain, which has structural similarity to Ras association or Ras binding domains [39]. This mediates enhanced MAPK signalling, while also promoting apoptosis through the p53-dependent pathway [23,27]. We recently showed that the GTP-H-ras specific, dimeric nanocluster scaffold Gal-1 increases MAPK-signalling, in a dimerization dependent fashion, by directly binding to the Ras binding domains of effectors, such as Raf [15]. We therefore hypothesized, that other oligomeric proteins that would bind to active Ras via a Ras binding domain-like domain could possess a similar nanocluster scaffolding activity.

**Fig 1:**
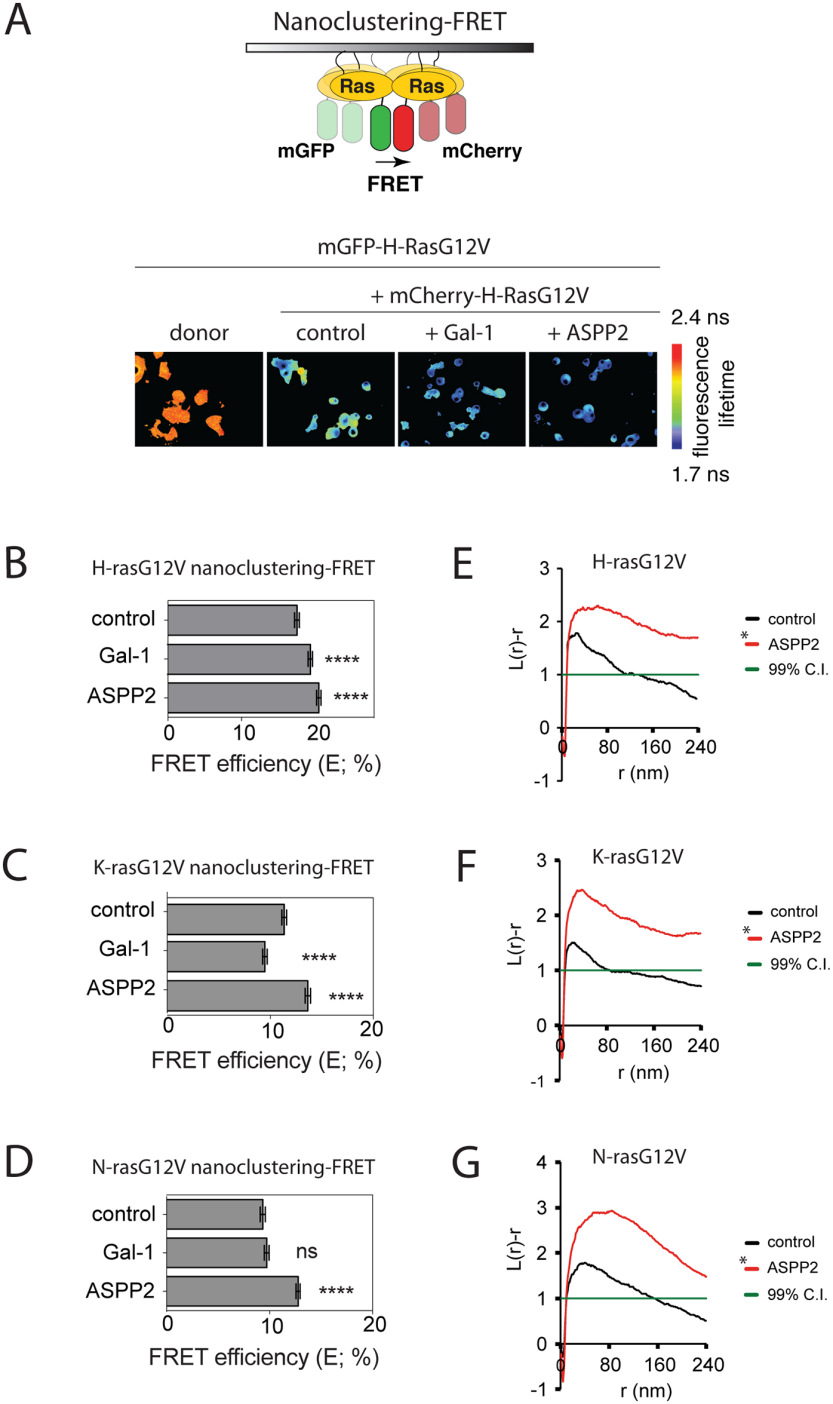
ASPP2 increases oncogenic H-ras, K-ras and N-ras nanoclustering. **(A)** *Top***,** scheme explaining nanoclustering-FRET analysis in HEK cells. Green and red ovals represent mGFP- and mCherry-tags, respectively. *Bottom*, examples of FLIM-FRET images of HEK cells from the different FRET samples as indicated. (**B-D**) Nanoclustering-FRET analysis in HEK cells coexpressing mGFP- and mCherry-tagged (**B**) H-rasG12V, (**C**) K-rasG12V or (**D**) N-rasG12V. The effect of Gal-1 or ASPP2 expression on nanoclustering-FRET was compared to control samples. Statistical significance of differences between controls and treated samples was examined using one-way ANOVA (mean ± SEM, n = 3; ns, not significant; *, p<0.05, ****, p< 0.0001). (**E-G**) Electron microscopic nanoclustering analysis of BHK cells expressing mGFP-tagged (**E**) H-rasG12V, (**F**) K-rasG12V or (**G**) N-rasG12V alone or together with ASPP2. mGFP was immunolabeled with 4.5 nm gold nanoparticles coupled to anti-GFP antibody. The spatial distribution of gold particles was evaluated using univariate K-function, where L(r)–r values indicate the extent of nanoclustering as a function of length scale, r, in nm. At least 15 images were analysed for each condition. Statistical significance between different conditions was evaluated using bootstrap tests. Averaged curves are shown for each condition.

In order to test for any effect of ASPP2 on Ras isoform specific nanoclustering, we used our well-established nanoclustering-FRET readout (Fig 1A) [14,40,41]. FRET pairs of mGFP- and mCherry-tagged RasG12V were expressed in HEK cells that either coexpressed Gal-1, as a positive control for H-ras nanoclustering, or ASPP2 (S1A Fig). Overexpression of either protein significantly increased H-ras nanoclustering (Fig 1B). However, ASPP2 also promoted increased K-ras nanoclustering, while Gal-1 showed its recently reported negative effect on K-ras nanoclustering [15,41] (Fig 1C). Of note, also N-ras nanoclustering was increased by ASPP2 overexpression, while we found no effect by Gal-1 expression (Fig 1D). Therefore ASPP2 is the first N-ras nanocluster scaffold.

The ability of ASPP2 to enhance Ras nanoclustering was also examined using electron microscopy (EM)-spatial mapping. Intact plasma membrane sheets of BHK cells expressing one of the mGFP-tagged Ras isoforms without/ with ASPP2 were attached to EM grids and immunolabeled with 4.5 nm gold nanoparticles coupled to an anti-GFP antibody. The spatial distribution of gold particles was analysed using Ripley’s K-function. In Fig 1E–1G the extent of nanoclustering, *L(r)-r*, was plotted against the cluster radius (*r*). *L(r)-r* values above the 99% confidence interval (99% C.I.) indicate statistically nanoclustering. As expected, ASPP2 significantly enhanced nanoclustering of all Ras isoforms (Fig 1E–1G), entirely consistent with the FLIM data.

### ASPP2 promotes effector recruitment and Ras-signalling of all Ras isoforms

**Fig 2:**
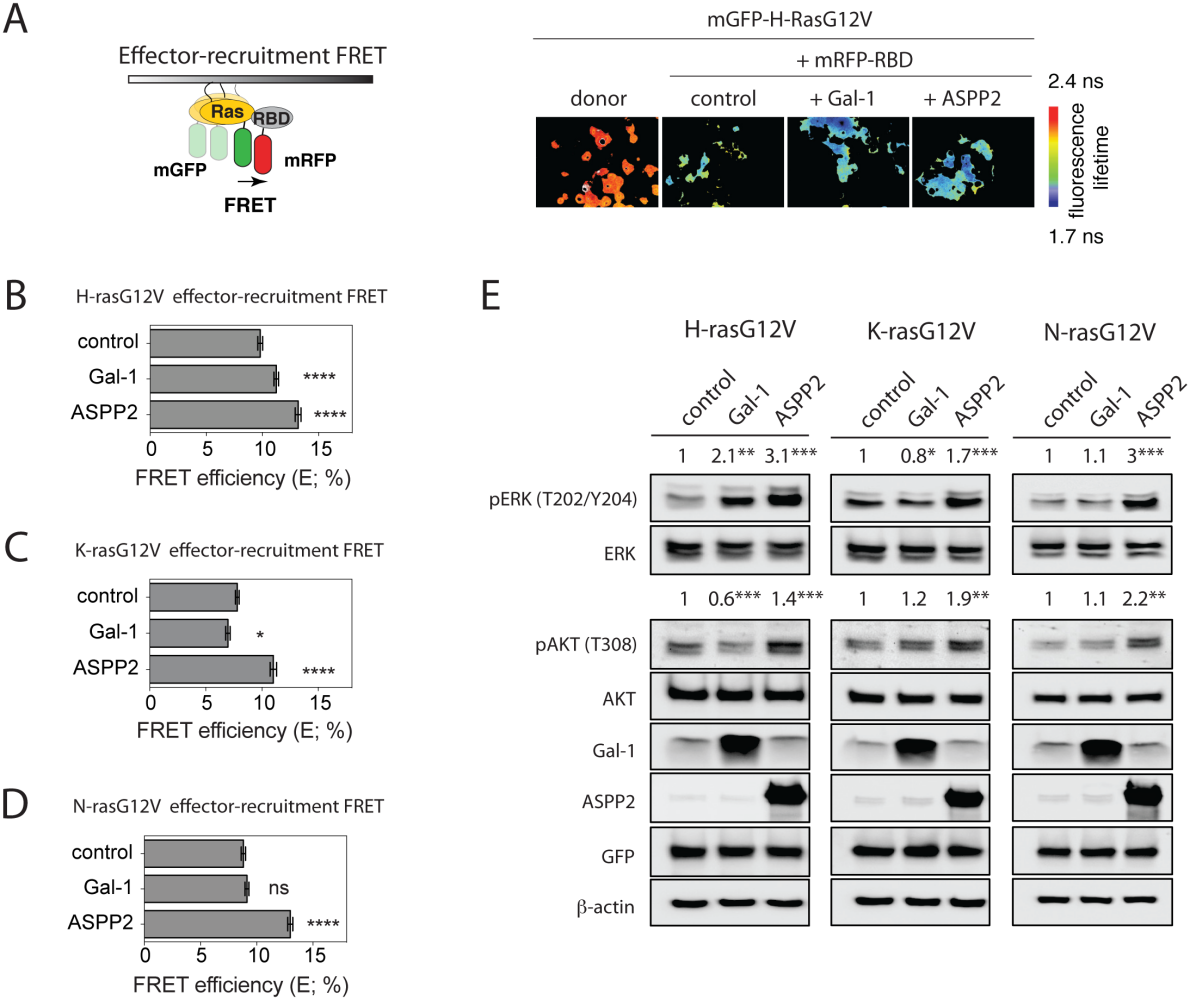
ASPP2 increases oncogenic H-ras, K-ras and N-ras-effector-recruitment, as well as ERK- and AKT-signalling. (**A**) *Left*, scheme explaining effector-recruitment FRET analysis in HEK cells. *Right*, examples of FLIM-FRET images of HEK cells from the different FRET samples as indicated. (**B-D**) Effector-recruitment FRET analysis in HEK cells coexpressing (**B**) mGFP-H-rasG12V, (**C**) mGFP-K-rasG12V or (**D**) mGFP-NrasG12V and mRFP-RBD from C-Raf. The effect of Gal-1 or ASPP2 expression on effector-recruitment FRET was compared to control samples. (**E**) Representative Western blots from HEK cells expressing mGFP-H-rasG12V (*left*), K-rasG12V (*middle*) or N-rasG12V (*right*) without or with Gal-1 or ASPP2. Statistical significance of differences between controls and treated samples was examined using one-way ANOVA (mean ± SEM, n = 3; ns, not significant; *, p<0.05, **, p< 0.01, ***, p<0.001, ****, p< 0.0001).

We previously established a cell-based Ras effector-recruitment FRET assay, using the mRFP-tagged Ras binding domain of C-Raf (RBD) and the mGFP-tagged RasG12V (Fig 2A). Using this assay, we showed that Gal-1 modulates effector recruitment in agreement with its effect on Ras nanoclustering [14] (Fig 2B–2D). Likewise, we observed that ASPP2 significantly stimulated RBD recruitment to active H-, K- or N-ras (Fig 2B–2D). In agreement with the general effector engagement, ASPP2 expression was also associated with a significant increase in downstream ERK and AKT signalling output of HEK cells expressing either of the oncogenic Ras isoforms (Fig 2E). Conversely, Gal-1 expression stimulated pERK and abrogated pAKT-levels in H-rasG12V expressing cells (Fig 2E, ***left***) as previously observed [21]. Moreover, it abrogated pERK in K-rasG12V expressing cells, while pAKT levels remained unaffected (Fig 2E, ***center***), but it had no effect on N-rasG12V associated signalling (Fig 2E, **right)**. Overall, for both ASPP2 and Gal-1, their effect on pERK levels correlated exactly with that on nanoclustering-FRET and effector-recruitment FRET.

In conclusion, these data suggest that ASPP2 is a novel, pan-Ras nanoclustering scaffold that enhances effector recruitment and activates downstream ERK and AKT signalling.

### N- and C-terminal ASPP2 truncation mutants are sufficient to induce Ras nanocluster enhancement

**Fig 3:**
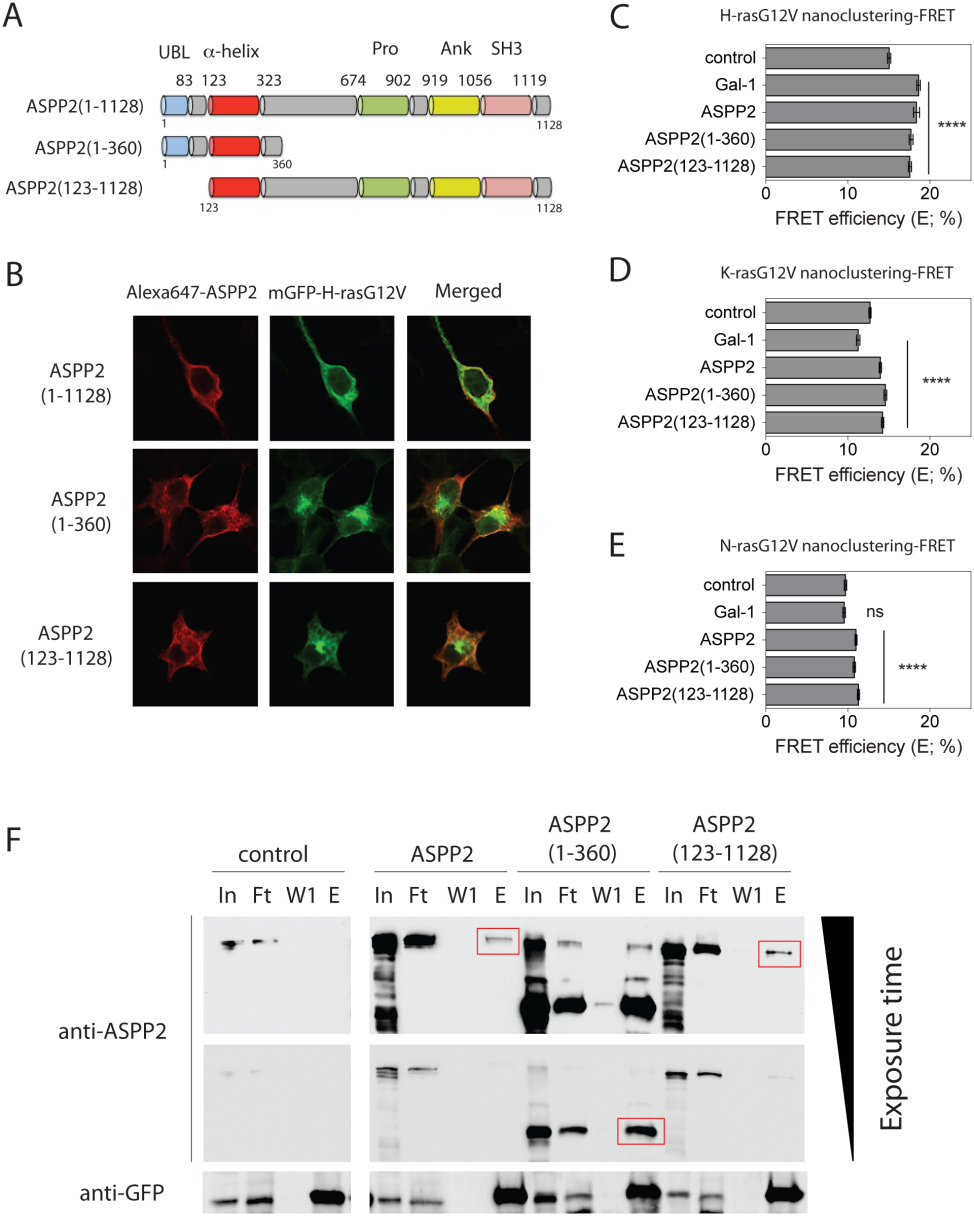
N- and C-terminal truncation mutants of ASPP2 can still promote Ras nanoclustering. (**A**) Schematic of full-length ASPP2, as well as ASPP2(1–360) and ASPP2(123–1128) truncation mutants. ASPP2 domains from left to right: Ubl, ubiquitin-like domain; α-helical domain; Pro, proline-rich domain; Ank, Ankyrin repeats; SH3, SRC homology 3 domain. (**B**) Confocal microscopic images of HEK cells cotransfected with mGFP-H-rasG12V (green) and full-length or truncated ASPP2 (red). (**C-E**) Nanoclustering-FRET analysis of HEK cells coexpressing mGFP- and mCherry-tagged (**C**) H-rasG12V, (**D**) K-rasG12V or (**E**) N-rasG12V. Cells were analysed after overexpression of Gal-1, full-length ASPP2 or its truncation mutants. (**C-E**) Statistical significance of differences between controls and treated samples was examined using one-way ANOVA (mean ± SEM, n = 3; ns, not significant; ****, p< 0.0001). (**F**) Western blot of anti-GFP immunoprecipitation samples probed with anti-ASPP2- (top) or anti-GFP- (bottom) antibodies. Samples were lysates prepared from mGFP-H-rasG12V transfected HEK cells that were cotransfected with full-length ASPP2 or its truncation mutants or an empty plasmid (control), as indicated. In, input; Ft, flow-through; W1, wash; E, elution. Red boxes indicate the immunoprecipitated ASPP2 fragments.

In order to understand, which domain of ASPP2 is responsible for increasing Ras nanoclustering we examined two truncation mutants (Fig 3A, S1B Fig). Neither truncation of the N-terminal 122 amino acids that comprise the Ubl domain of ASPP2, nor that of the C-terminal fragment downstream of residue 360, which includes the proline-rich-, ankyrin- and SH3-domains, affected the localization of ASPP2-constructs or Ras isoforms if coexpressed in HEK cells (Fig 3B, S2A–S2C Fig). Consistent with previous observations on full length ASPP2 and H-ras [27], both protein fragments partially colocalized at the plasma membrane with H-, K- and N-ras (Fig 3B, S2B and S2C Fig). Note that, subcellular localization and nanoclustering-FRET results obtained with full-length ASPP2 encoded by pcDNA3-ASPP2(1–1128)-V5, which has the same backbone as the ASPP2 truncation mutants, were similar to those obtained with full-length ASPP2 expressed from pCMV-Sport6-ASPP2 (data not shown).

Interestingly, these truncations did not affect the ability of ASPP2 to act as a general nanocluster-promoting scaffold for active Ras proteins (Fig 3C–3E), while inactive Ras nanoclustering was not affected (S2D Fig). This was supported by immunoprecipitation experiments, which confirmed that truncation mutants retained their ability to interact with active Ras (Fig 3F). Hence, these data point to the α-helix as an essential domain for mediating direct or indirect Ras interaction and Ras nanoclustering enhancement.

### ASPP2 blocks Gal-1 dependent nanoclustering and transforming ability of oncogenic H-ras

Given that Gal-1 promotes cell proliferation and transformation [19], while ASPP2 induces apoptosis or senescence [23], we wondered whether these two proteins would compete on the nanoscale for Ras proteins. As Gal-1 does not affect N-ras nanoclustering, we focused on H- and K-ras only. In addition, we continued working only with the pCMV-Sport6-ASPP2 plasmid because the expression of full-length ASPP2 from this plasmid was similar to the expression of Gal-1 (S1B Fig).

**Fig 4:**
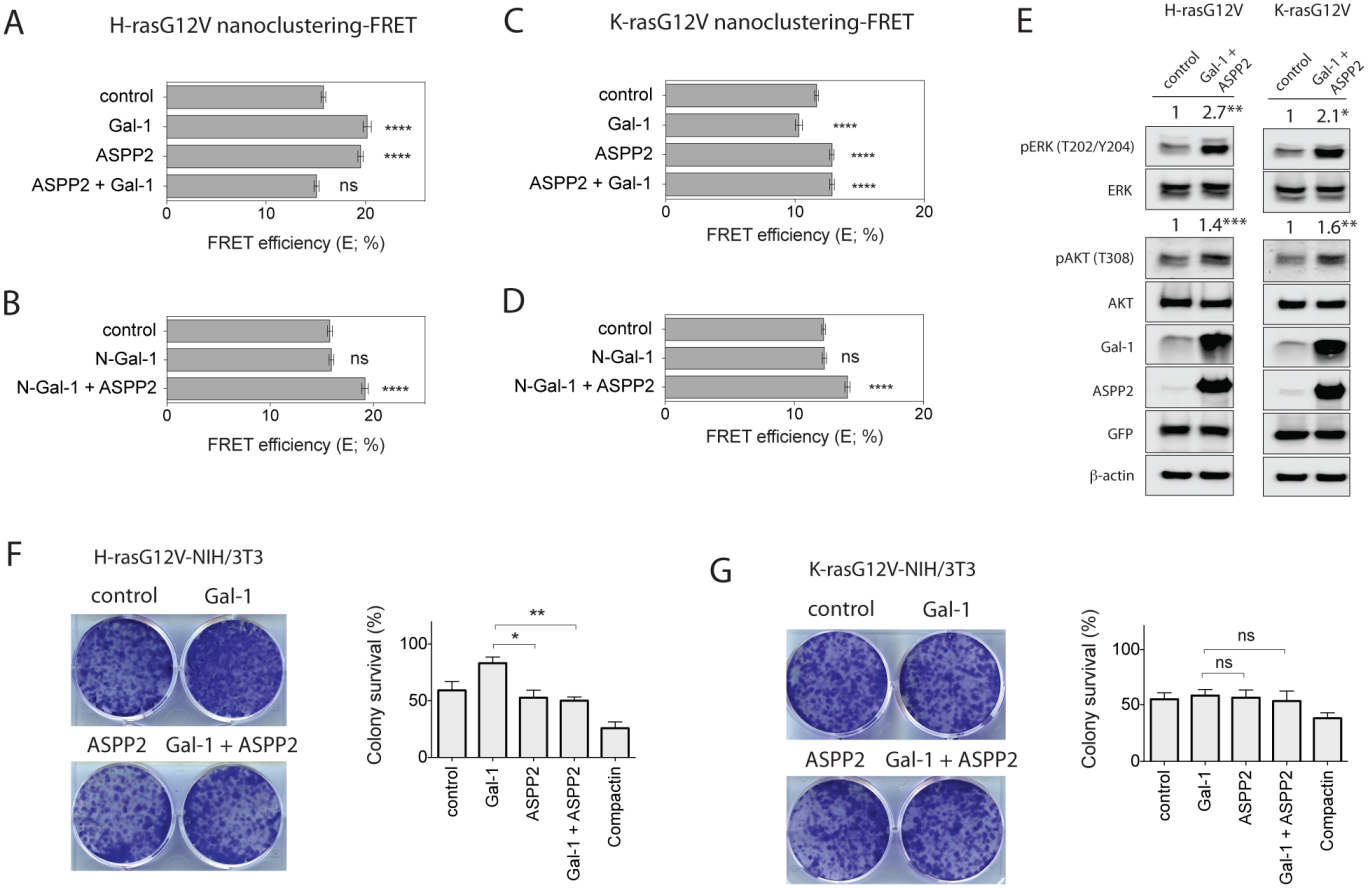
ASPP2 blocks Gal-1 dependent nanoclustering and halts oncogenic H-ras induced transformation. Nanoclustering-FRET analysis in HEK cells coexpressing mGFP- and mCherry-tagged (**A, B**) H-rasG12V or (**C, D**) K-rasG12V. Cells were analysed after overexpression of either Gal-1 or ASPP2 plasmids, or both (1:1 ratio). Plotted are the means ± SEM, n = 3. (**E**) Representative Western blots from HEK cells expressing mGFP-H-rasG12V (*left*) or K-rasG12V (*right*) alone or together with Gal-1 and ASPP2. Statistical significance of differences was examined using t-test (n = 3; *, p<0.05, **, p< 0.01, ***, p<0.001). (**F, G**) Colony survival assay of NIH/3T3 cells stably expressing (**F**) H-rasG12V or (**G**) K-rasG12V and transiently expressing indicated constructs. Colony survival was graphed based on mean foci areas calculated from at least 4 independent biological repeats. (**A-D, F-G**) Statistical significance of differences between controls and treated samples was examined using one-way ANOVA (ns, not significant; *, p<0.05; **, p<0.01; ****, p<0.0001).

As before, we coexpressed in HEK cells our FRET pairs of mGFP- and mCherry-tagged H-rasG12V (Fig 4A and 4B) or K-rasG12V (Fig 4C and 4D) together with Gal-1, ASPP2, or of both nanocluster scaffold proteins coexpressed together. Intriguingly, the H-rasG12V nanoclustering-FRET increase was completely abolished when both Gal-1 and ASPP2 were expressed together (Fig 4A), suggesting that both proteins cancel each other’s nanocluster promoting activity out. This also applied to the N- and C-terminal truncation mutants of ASPP2 (S3A Fig). However, a dimerization-deficient mutant of Gal-1 (N-Gal-1) that does not support H-ras nanoclustering [15], was not able to compete for the nanocluster promoting activity of ASPP2 (Fig 4B).

In the case of K-rasG12V, both full-length and the truncated ASPP2 proteins were able to revert the inhibitory effect of Gal-1 on K-ras nanoclustering-FRET, thus acting dominantly to promote K-rasG12V clustering increase (Fig 4C, S3B Fig). Again, N-Gal-1 did not affect the ASPP2 induced increase in K-ras nanoclustering (Fig 4D). In agreement with ASPP2 outcompeting Gal-1 nanoclustering effects (Fig 4A and 4C), pERK and pAKT levels of H-rasG12V (Fig 4E**, *left***) or K-rasG12V (Fig 4E, ***right***) transfected HEK cells were the same as those of cells transfected with ASPP2 alone.

In the light of the different activities of Gal-1 and ASPP2 on cell growth [19,27], we were interested in the functional consequences of their antagonistic effects on nanoclustering in transformed cells. First we used NIH/3T3 cells that were stably transformed with either H-rasG12V or K-rasG12V [13]. Due to the transient expression of Gal-1 and ASPP2 in these cells, we studied their effect after first colonies were formed [37]. In agreement with what we observed for H-ras nanoclustering, Gal-1 expression increased colony survival of NIH/3T3 transformed with H-rasG12V, whereas coexpression with ASPP2 neutralized this Gal-1 effect. However, expression of ASPP2 on its own had no significant influence on colony survival (Fig 4F). K-rasG12V expressing NIH/3T3 cells did not exhibit any significant changes in colony survival by transfection of Gal-1, ASPP2 or both combined (Fig 4G). Thus the ASPP2 mediated increase in nanoclustering of either H-rasG12V or K-rasG12V and downstream signalling does not significantly affect colony survival. However, in agreement with the nanoclustering data, the Gal-1 activity is neutralized by ASPP2 coexpression in cells expressing oncogenic H-rasG12V.

### Gal-1 cannot override ASPP2 induced senescence and inhibition of tumourosphere formation

**Fig 5:**
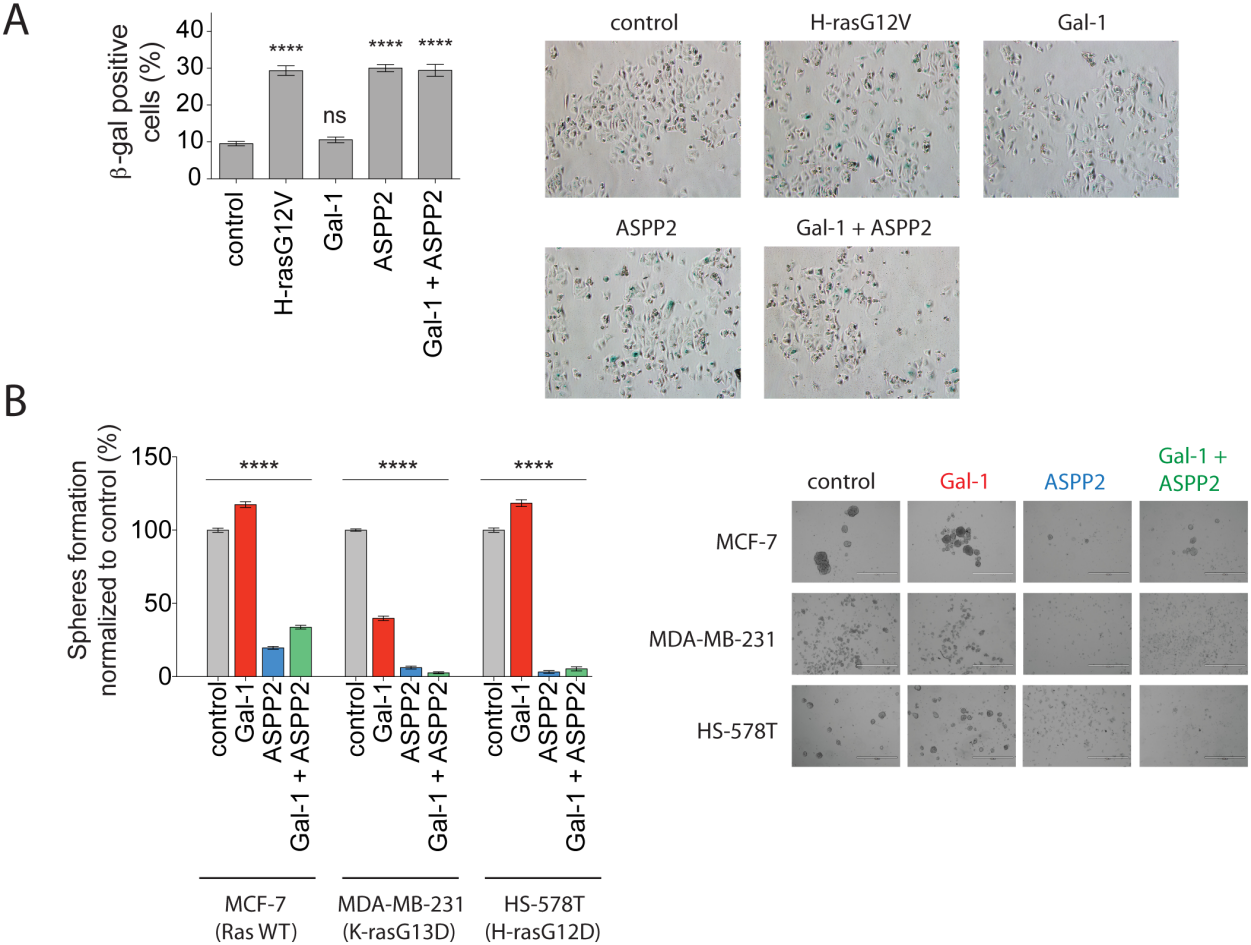
ASPP2 dominates over Gal-1 thus robustly inducing senescence and abrogating mammosphere formation. (**A**) SA-β-gal assay of MCF-7 cells transfected with plasmids encoding H-rasG12V, ASPP2, Gal-1 or the combination of the latter two, as indicated. Cells were stained 7 days after transfection. On the left, percentages of SA-β-gal positive cells are shown in the graph (mean ± SEM, n = 3). On the right, representative images from the assay. (**B**) Mammosphere formation assay with MCF-7, MDA-MB-231 or HS-578T breast cancer cell lines. Mammospheres were transfected with Gal-1, ASPP2, or both (1:1 ratio) and cells were then grown under non-adherent conditions for 9 days. On the right, representative images of mammospheres are shown as indicated. (**A, B**) Statistical significance of differences between controls and treated samples was examined using one-way ANOVA (mean ± SEM n≥3; ns, not significant; ****, p<0.0001).

ASPP2 mediates oncogenic H-ras induced senescence [27]. We therefore investigated whether Gal-1 can antagonize this ASPP2 activity (Fig 5A). We expressed Gal-1, ASPP2 or both combined in MCF-7 breast cancer cells and then quantified senescent cells with β-galactosidase staining 7 days after transfection. Overexpression of H-rasG12V served as a positive control for senescence induction [1,27] As expected, H-rasG12V or ASPP2 expression induced senescence, while Gal-1 expression alone did not increase cell senescence above control levels. When Gal-1 and ASPP2 were coexpressed, senescence was induced to the same extent as by ASPP2 expression alone (Fig 5A).

Finally, in view of the emerging role of the ASPP members in stem cell regulation [26], we examined whether ASPP2 might affect stemness properties of cancer cells. Mammospheres possess increased tumour seeding potential and are resistant against classical cancer drugs [42,43]. Therefore, mammosphere formation is considered to report on essential features of cancer stem cells [44]. Similar to what we observed for NIH/3T3 cells, mammosphere formation of H-rasG12D and p53 mutated HS-578T, but also of Ras and p53 wt MCF-7 was promoted by Gal-1 alone (Fig 5B). By contrast, sphere formation of K-rasG13D and p53 mutated MDA-MB-231 was significantly abrogated by Gal-1 expression, which was in agreement with the negative effect of Gal-1 on K-ras nanoclustering (Fig 1C). If we then expressed ASPP2 alone or in combination with Gal-1, we completely abrogated sphere formation in Ras-mutated HS-578T and MDA-MB-231, while it was significantly decreased in MCF-7.

In conclusion, ASPP2 dominates over the pro-tumourigenic effects of Gal-1, thus robustly inducing senescence and abrogating mammosphere formation. This dominance is particularly obvious in H-ras mutated or Ras wt breast cancer cell lines, where Gal-1 expression alone significantly promotes sphere formation.

## Discussion

ASPP2 interacts with Ras and p53 proteins thus literally being at the crossroad of drivers and suppressors of tumour growth, respectively [27,45]. Here we examined, whether ASSP2 displays any Ras isoform specificity and how it engages with the Ras membrane signalling system on the nanoscale.

We showed that ASPP2 increases nanoclustering of active H-ras, K-ras and N-ras thus being the first pan-Ras nanocluster scaffold. Our data with C- and N-terminal deletion mutants of ASPP2 suggest that the α-helical domain is important for this activity. Like Gal-1, a known nanocluster scaffold, ASPP2 potentiates effector recruitment to Ras and enhances Ras downstream signalling. Intriguingly, ASPP2 competes with the tumour promoting nanocluster scaffold Gal-1, which positively regulates GTP-H-ras nanocluster. Thus ASPP2 efficiently diverts Ras signalling to cellular senescence or blockage of stemness features of breast cancer cell lines. We therefore tentatively suggest that ASPP2 acts as a janus-faced gatekeeper of GTP-Ras, on the one hand promoting nanoclustering, effector recruitment, MAPK- and AKT-signalling, but on the other hand directly linking the overactivity of this pathway to apoptosis and senescence to keep its transforming and tumourigenic potential in check.

Others previously reported that the Ubl domain of ASPP2 was required for Ras interaction [27,39]. However, our results suggest that Ras-interaction and nanocluster promotion converge on the α-helical domain of ASPP2 (Fig 3C–3F). Our recently published data suggest that Gal-1 promotes homo- or heterodimerization of B-Raf and A-Raf to increase GTP-H-ras nanoclustering [15]. In conjunction with recent evidence for Ras dimers [46,47], we proposed stacked dimers of H-ras::B/A-Raf::Gal-1 in a nanoclustered signalling unit [15]. Therefore it is particularly interesting that ASPP2 is also able to promote Raf dimerization, however of B- and C-Raf [27]. This could present a mechanism of how ASPP2 dominates over Gal-1 on the nanoscale (Fig 4A and 4C), namely by more efficiently segregating B-Raf into B-Raf containing dimers. This segregation might be consolidated in conjunction with Ras by ASPP2’s ability to bind Raf and Ras simultaneously [23], while Gal-1 does not interact directly with H-ras [15]. In conclusion, we propose that either nanocluster scaffold protein, Gal-1 or ASPP2, integrates into stacked dimers of Ras::Raf::Gal-1 or ASPP2.

While both Gal-1 and ASPP2 increase nanoclustering (Fig 1B–1G) and effector recruitment (Fig 2B–2D), the cellular outcome is the exact opposite, with ASPP2 dominantly driving senescence or abolishing mammosphere formation (Fig 5). This dominance of ASPP2 is partly reflected on the nanoscale (Fig 4A and 4C) and by its signalling response prevailing over that of Gal-1 (Fig 4E as compared to Fig 2E). However, the increased nanoclustering does not conclusively report on the remodelling of the Ras-associated signalling complexes. These nanocluster scaffold instructed complexes must somehow determine the overall different, partly Ras isoform-specific pERK- and pAKT-responses. However, we consistently observed a very good correlation of nanoclustering with pERK in this and other studies [13,38,41]. Moreover, the different cellular outcome that is observed with ASPP2 as compared to Gal-1, is likely associated with the very different protein domain composition and consequently biological interactions, notably that with p53 [3].

Furthermore, we confirmed that ASPP2 can abrogate cell growth in 3D spheres even in p53-mutated cancer cells (Fig 5B), consistent with its p53-independent activities that were reported previously [48]. Of similar interest, ASPP2 can suppress the tumorigenic potential of breast cancer cells irrespective of the Ras-mutation status. The high efficiency with which ASPP2 can realize this is probably due to its ability to engage with all oncogenic Ras isoforms.

Our data suggest a revised mechanism of how ASPP2 converts oncogenic Ras activity efficiently into tumour growth suppression. ASPP2 engages with any Ras isoform to enhance nanoscale signalling complexes thus promoting activation of the major Ras-signalling pathways, including the MAPK-pathway that is required for its translocation to the nucleus and engagement with p53 to execute apoptosis or senescence [3]. Importantly, ASPP2 has the ability to outcompete the tumour promoter Gal-1 in nanoscale signalling complexes of membrane anchored Ras.

## Supporting Information

S1 Fig Analysis of protein expression by Western blot. **(A)** Representative Western blots (n>10) from HEK cells expressing ASPP2- or Gal-1-plasmids (indicated on top). Probing antibodies are shown to the left. (**B**) Representative anti-ASPP2 Western blot (n>10) from HEK cells transfected with pCMV-Sport6-ASPP2 (lane 2), pcDNA3-ASPP2(1–1128)-V5 (lane 3), pcDNA3-ASPP2(123–1128)-V5 (lane 4) and pcDNA3-ASPP2(1–360)-V5 (lane 5).
(TIF)

S2 Fig ASPP2 truncation mutants localize similarly to full-length ASPP2. (**A**) Confocal microscopic images of HEK cells transfected with mGFP-RasG12V (green) isoforms or the full-length and truncated ASPP2 (red). (**B-C**) Confocal fluorescence microscopy on HEK cells cotransfected with (**B**) mGFP-K-rasG12V or (**C**) mGFP-N-rasG12V (green) and full-length or truncated ASPP2 (red). (**D**) Nanoclustering-FRET analysis in HEK cells coexpressing mGFP- and mCherry-tagged wild-type H-ras. Cells were serum-starved for 6 hours after overexpression of Gal-1 or the ASPP2 proteins. Statistical significance of differences between control and treated samples was examined using one-way ANOVA (ns, not significant).
(TIF)

S3 Fig ASPP2 truncation mutants block Gal-1 dependent nanoclustering. (**A-B**) Nanoclustering-FRET analysis in HEK cells coexpressing mGFP- and mCherry-tagged (**A**) H-rasG12V or (**B)** K-rasG12V. Cells were analysed after overexpression with the proteins as indicated (mean ± SEM, n = 3). Statistical significance of differences between control and treated samples was examined using one-way ANOVA (ns, not significant; *, p<0.05; **, p<0.01; ****, p<0.0001).
(TIF)
