# Supplementary figures and images for: ASPP2 Is a Novel Pan-Ras Nanocluster Scaffold
Source: PLoS One. 2016 Jul 20;11(7):e0159677. doi: 10.1371/journal.pone.0159677 (PMC4954646; doi:10.1371/journal.pone.0159677)

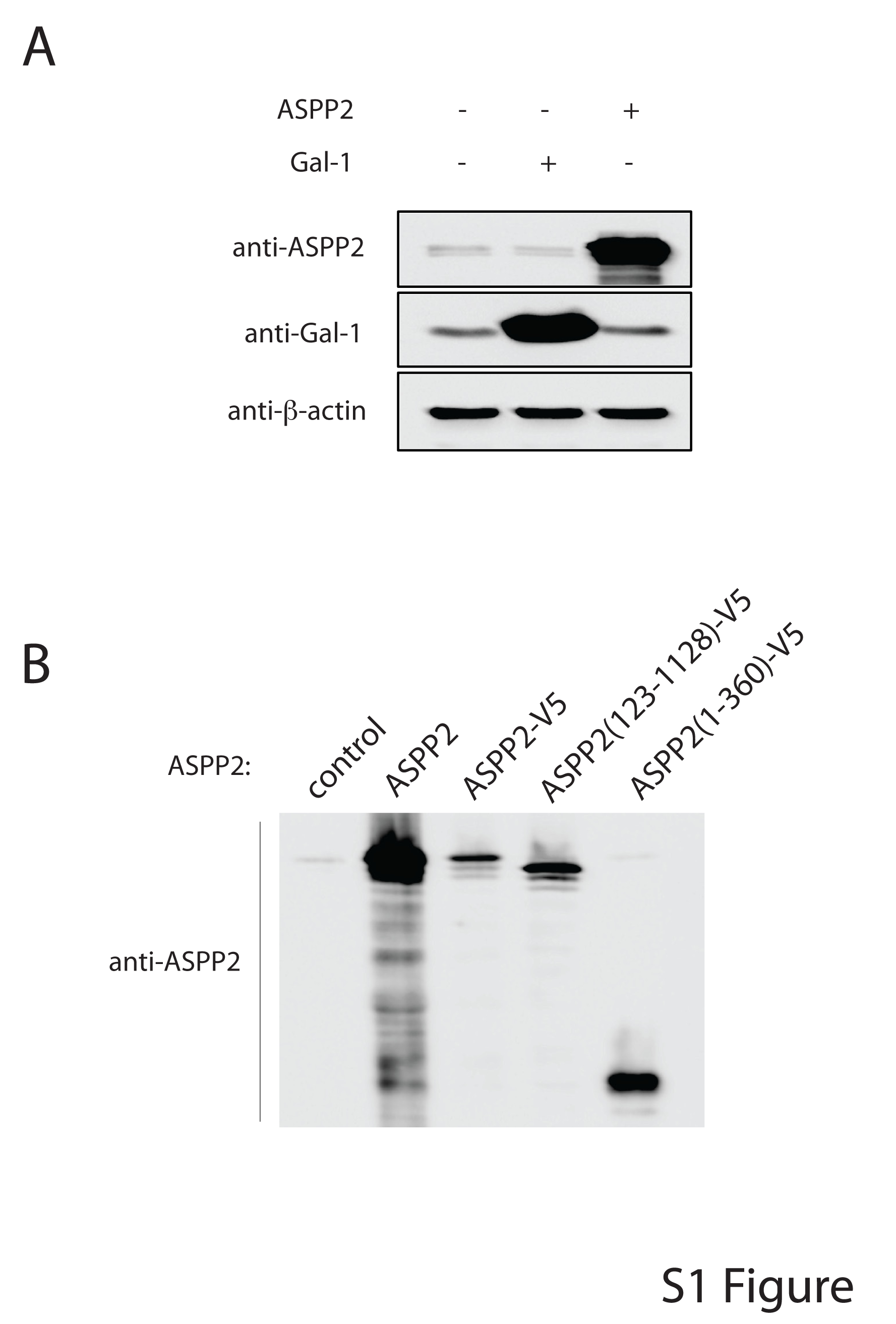

Supplement: S1 Fig — (A) Representative Western blots (n>10) from HEK cells expressing ASPP2- or Gal-1-plasmids (indicated on top). Probing antibodies are shown to the left. (B) Representative anti-ASPP2 Western blot (n>10) from HEK cells transfected with pCMV-Sport6-ASPP2 (lane 2), pcDNA3-ASPP2(1–1128)-V5 (lane 3), pcDNA3-ASPP2(123–1128)-V5 (lane 4) and pcDNA3-ASPP2(1–360)-V5 (lane 5). (TIF) [file pone.0159677.s001.tif]

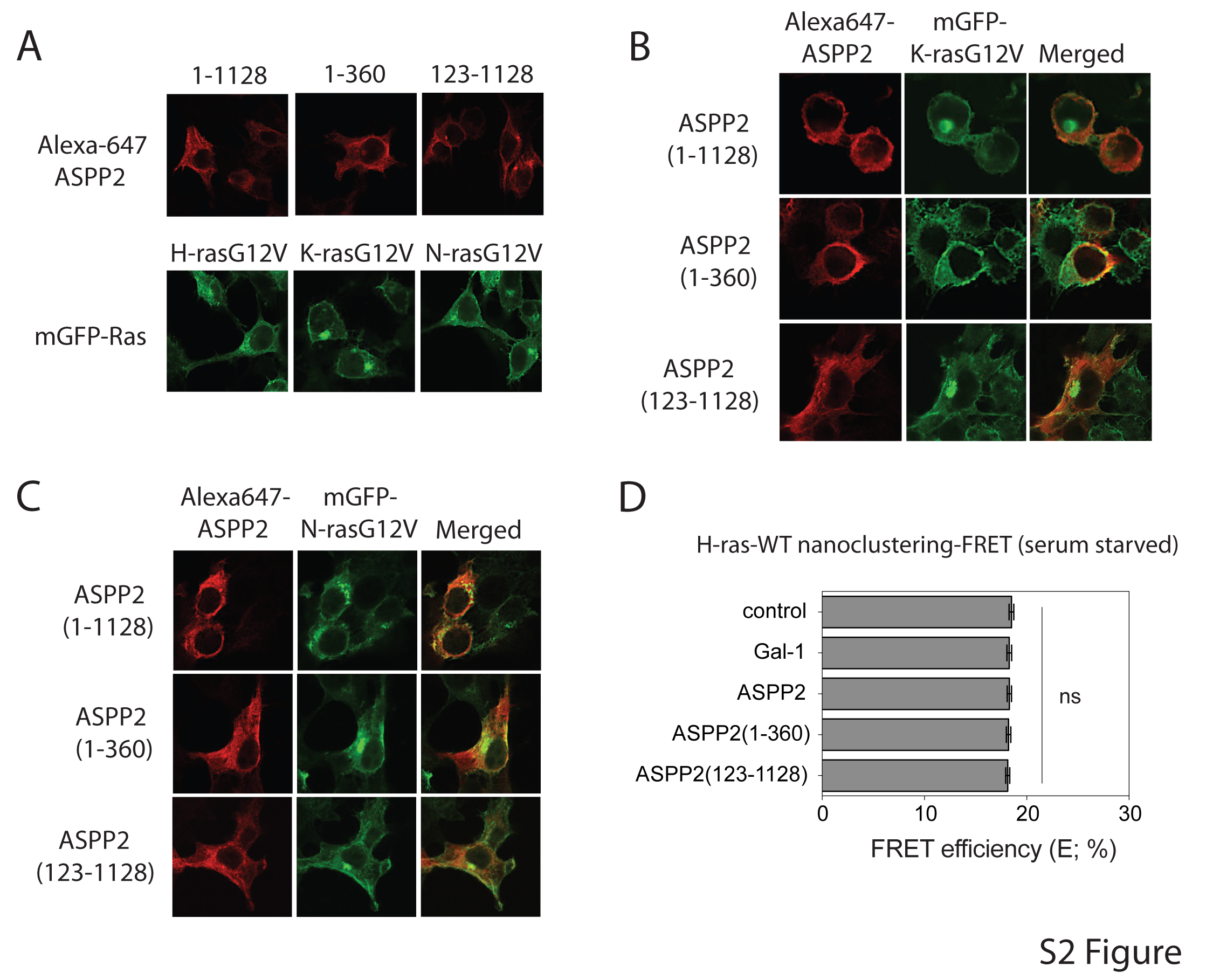

Supplement: S2 Fig — (A) Confocal microscopic images of HEK cells transfected with mGFP-RasG12V (green) isoforms or the full-length and truncated ASPP2 (red). (B-C) Confocal fluorescence microscopy on HEK cells cotransfected with (B) mGFP-K-rasG12V or (C) mGFP-N-rasG12V (green) and full-length or truncated ASPP2 (red). (D) Nanoclustering-FRET analysis in HEK cells coexpressing mGFP- and mCherry-tagged wild-type H-ras. Cells were serum-starved for 6 hours after overexpression of Gal-1 or the ASPP2 proteins. Statistical significance of differences between control and treated samples was examined using one-way ANOVA (ns, not significant). (TIF) [file pone.0159677.s002.tif]

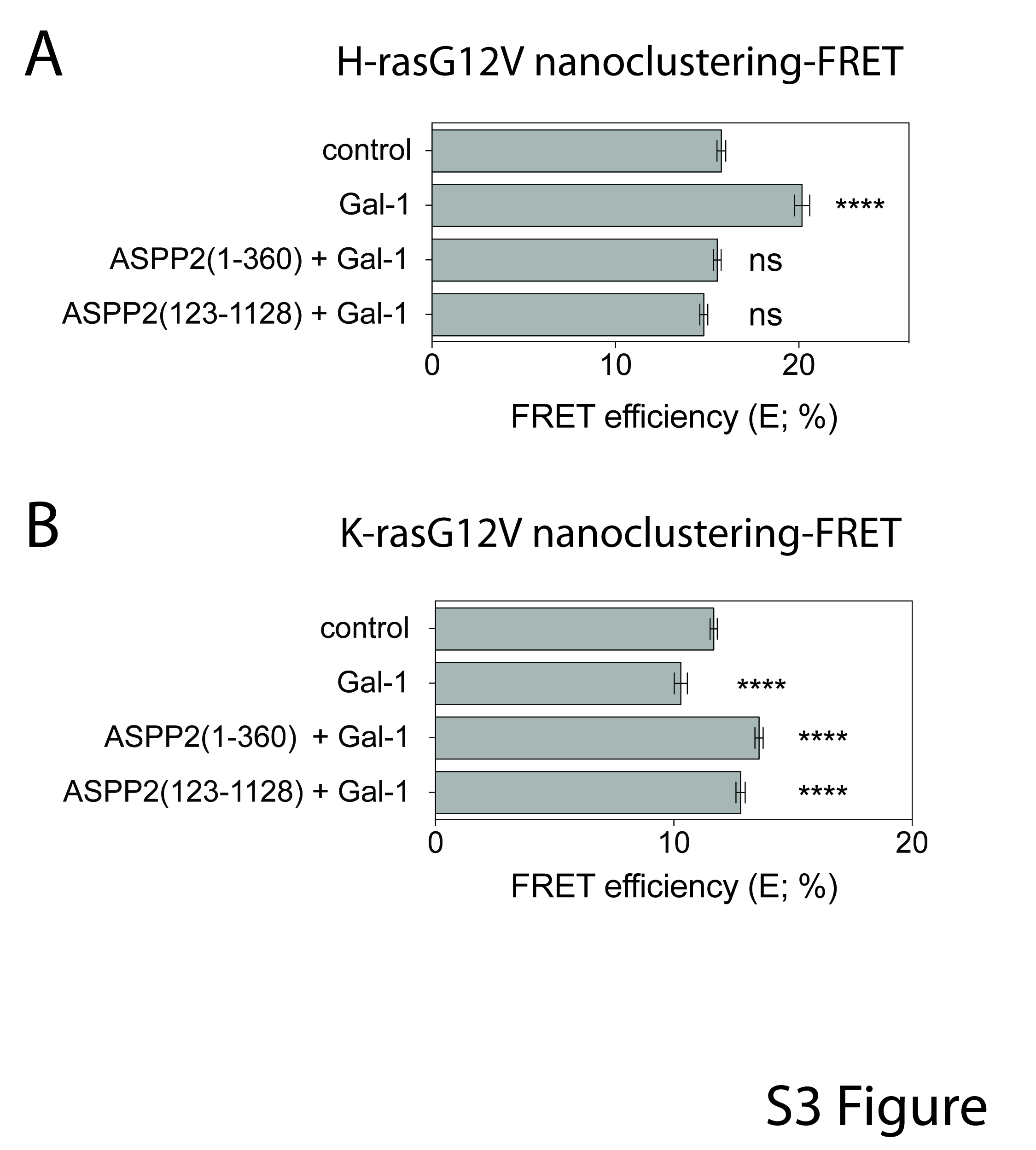

Supplement: S3 Fig — (A-B) Nanoclustering-FRET analysis in HEK cells coexpressing mGFP- and mCherry-tagged (A) H-rasG12V or (B) K-rasG12V. Cells were analysed after overexpression with the proteins as indicated (mean ± SEM, n = 3). Statistical significance of differences between control and treated samples was examined using one-way ANOVA (ns, not significant; *, p<0.05; **, p<0.01; ****, p<0.0001). (TIF) [file pone.0159677.s003.tif]
